# Editorial: Polyphenol-based dietary intervention against neurodegenerative disorders

**DOI:** 10.3389/fnut.2023.1208308

**Published:** 2023-08-14

**Authors:** Farhan Saeed, Muhammad Imran, Ghulam Hussain, Hafiz A. R. Suleria, Ali Imran

**Affiliations:** ^1^Department of Food Science, Faculty of Life Sciences, Government College University, Faisalabad, Pakistan; ^2^Department of Food Science, University of Narowal, Narowal, Pakistan; ^3^Neurochemicalbiology and Genetics Laboratory (NGL), Department of Physiology, Faculty of Life Sciences, Government College University, Faisalabad, Pakistan; ^4^School of Agriculture, Food and Ecosystem Sciences, Faculty of Science, The University of Melbourne, Parkville, VIC, Australia

**Keywords:** polyphenols, oxidative stress, neuroprotective effect, dietary intervention, antioxidants

The articles in this special Research Topic entitled “*Polyphenol-based dietary intervention against neurodegenerative disorders*” elucidate the neuroprotective effect of bioactive molecules-based dietary interventions. The primary focus has been paid to apprehending the role of oxidative stress in the pathogenesis of nerve disorders. This issue encompasses the four research articles accepted after various rounds of the peer review process. The factors responsible for acceptance were novelty, depth of study, completeness of experiment, and impact of the results. The first accepted article was submitted by Pratiwi et al.. They tested the therapeutic potential of stigmasterol, a phytosterol in nature against oxidative stress-mediated neuronal cell death, which is considered the leading cause of Alzheimer's disease (AD). In this article, the choice of material was exceptional as stigmasterol showed a tendency to cross the blood-brain barrier essential for a dietary component to be a neuroprotective agent. Moreover, the outcomes delineated that the induction of oxidative stress caused cell death that was curtailed significantly by the treatment of stigmasterol. Among the different mechanistic routes, upregulation of forkhead box O (FoxO) 3a, catalase, and anti-apoptotic protein B-cell lymphoma 2 (Bcl-2) in the neurons was found to be the major pathways for this effect.

Another work in this Research Topic by Ruankham et al. expounded on the modulatory effects of alpha-mangostin mediated by sirt1/3-foxo3a pathway in oxidative stress-induced neuronal cells. The significant findings of this article highlighted the active role of dietary intervention against oxidative stress-mediated neurodegenerative disorder under certain conditions. They wanted to explore the underlying molecular mechanism to explain the neuroprotective effect of alpha-mangostin in defending hydrogen peroxide (H_2_O_2_)-induced neurotoxicity. They adopted a systemic approach by using cytotoxicity, generation of ROS, and apoptosis, followed by protein expression profiling and molecular docking. They inferred that alpha-mangosteen could tackle oxidative stress that was found to deteriorate neural health by activating the SIRT1/3-FOXO3a pathway. However, they also highlight various rational limitations of this study, such that neuronal behaviors are limited to cell-cell interaction, tissue architecture, and intracellular microenvironments. They further emphasized that further *in vivo* studies still need to experimentally validate the antioxidant for pharmacokinetics in the body before clinical trials.

The 3^rd^ article of this issue by Wang et al. provides insight into the antioxidative and neuroprotective ability of flavonoids from Sea Buckthorn (TFH). They assessed the neuroprotective effect of TFH through the estimation of acetylcholinesterase (AChE) and monoamine oxidase A (MAO-A) activity in *Caenorhabditis elegans (C. elegans)*. The outcomes expounded that the TFH significantly delayed paralysis in *C. elegans* CL4176 and showed the potential of TFH as a promising agent to extend aging and treat neurodegenerative diseases. However, their study lacks in-depth mechanistic analysis and molecular mechanism and is advised for complete profiling of TFH to understand their most effective fraction to exert this beneficial impact.

The last article depicts the role of polyphenols and a natural phytochemical-enriched diet in managing migraine and associated complications by Bakırhan et al.. They observed a significant link between dietary patterns and migraine prognosis via mechanisms like systemic inflammation, vasodilation, cerebral glucose metabolism, and mitochondrial dysfunction. They involved patients with signature symptoms of migraine and under-treatment by a neurologist. They observed a negative correlation between migraine severity with the intake of phytochemicals and good diet quality. Their study is valuable for providing practical dietary guidelines to migraine patients. However, I believe the sample size should be widened to correlate this impact on a larger scale.

Conclusively, dietary polyphenols and phytochemicals may prove effective in managing the neurodegenerative disorders initiated by the cascade of phenomena mediated by oxidative stress. They can modulate various biomarkers involved in the pathogenesis of these diseases. However, their effective dose, metabolism, drug interaction, course of treatment and molecular mechanism are still lacking, which demands further intensive clinical trials to unveil the mechanistic concerns with these bioactive moieties.

## Author contributions

FS, MI, and GH provide idea and write-up guidelines. AI prepared the document. HARS provided the mentor role for this article. All authors contributed to the article and approved the submitted version.

